# Bis[5-(pyridin-2-yl)pyrazine-2-carbo­nitrile-κ^2^ *N* ^4^,*N* ^5^]silver hexa­fluorido­phosphate

**DOI:** 10.1107/S1600536811051403

**Published:** 2011-12-03

**Authors:** Zi Jia Wang

**Affiliations:** a Department of Chemistry, Capital Normal University, Beijing 100048, People’s Republic of China

## Abstract

In the mononuclear title complex, [Ag(C_10_H_6_N_4_)_2_]PF_6_, two κ^2^ *N*,*N*′-chelating 5-(pyridin-2-yl)pyrazine-2-carbonitrile ligands surround the Ag^I^ atom, forming a distorted N_4_ tetra­hedral coordination geometry. The mononuclear units are inter­connected through π–π inter­actions [centroid–centroid distances = 3.801 (2) and 3.979 (3) Å] and the hexa­fluoridophosphate anions are embedded within the inter­stices. C N⋯π inter­actions [N⋯centroid = 3.519 (2) Å] and C—H.⋯N hydrogen-bonding inter­actions also occur.

## Related literature

For coordination complexes with pyridyl-based ligands, see: Boudalis *et al.* (2003 ▶); Dunne *et al.* (1997 ▶); Huang *et al.* (2007 ▶); Wang *et al.* (2009 ▶). For a related complex with 5-(2-pyrid­yl)pyrazine-2-carbonitrile, see: Wang *et al.* (2010 ▶).
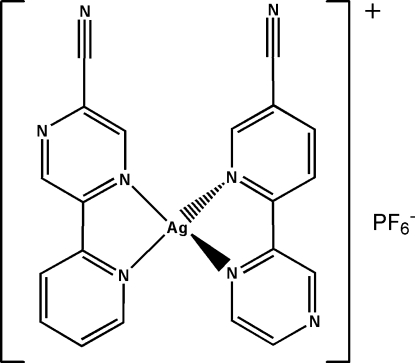


## Experimental

### 

#### Crystal data

[Ag(C_10_H_6_N_4_)_2_]PF_6_
*M* *_r_* = 617.22
Triclinic, 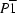

*a* = 8.8989 (9) Å
*b* = 9.1711 (10) Å
*c* = 14.0804 (15) Å
α = 77.023 (2)°
β = 86.926 (2)°
γ = 84.809 (2)°
*V* = 1114.5 (2) Å^3^
*Z* = 2
Mo *K*α radiation
μ = 1.05 mm^−1^
*T* = 293 K
0.38 × 0.30 × 0.30 mm

#### Data collection

Bruker APEXII CCD area-detector diffractometer
Absorption correction: multi-scan (*SADABS*; Bruker, 2007 ▶) *T*_min_ = 0.861, *T*_max_ = 1.000
8106 measured reflections
5438 independent reflections
4544 reflections with *I* > 2σ(*I*)
*R*_int_ = 0.017

#### Refinement

*R*[*F* ^2^ > 2σ(*F* ^2^)] = 0.032
*wR*(*F* ^2^) = 0.090
*S* = 1.04
5438 reflections
325 parameters
H-atom parameters constrained
Δρ_max_ = 0.71 e Å^−3^
Δρ_min_ = −0.37 e Å^−3^

### 

Data collection: *APEX2* (Bruker, 2007 ▶); cell refinement: *SAINT* (Bruker, 2007 ▶); data reduction: *SAINT*; program(s) used to solve structure: *SHELXS97* (Sheldrick, 2008 ▶); program(s) used to refine structure: *SHELXL97* (Sheldrick, 2008 ▶); molecular graphics: *SHELXTL* (Sheldrick, 2008 ▶); software used to prepare material for publication: *SHELXTL* and *PLATON* (Spek, 2009 ▶).

## Supplementary Material

Crystal structure: contains datablock(s) I, global. DOI: 10.1107/S1600536811051403/bt5734sup1.cif

Structure factors: contains datablock(s) I. DOI: 10.1107/S1600536811051403/bt5734Isup2.hkl

Additional supplementary materials:  crystallographic information; 3D view; checkCIF report

## Figures and Tables

**Table 1:** Hydrogen-bond geometry (Å, °)

*D*—H⋯*A*	*D*—H	H⋯*A*	*D*⋯*A*	*D*—H⋯*A*
C11—H11*A*⋯N7^i^	0.93	2.47	3.201 (2)	135

Symmetry code: (i) 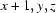.

**Fig. 1.**
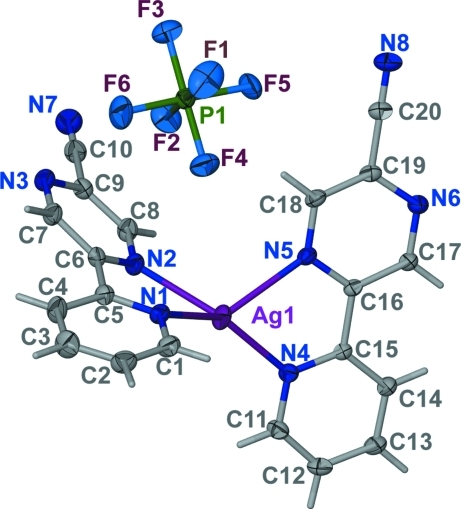
The atom-numbering scheme of the title compound with displacement ellipsoids drawn at the 30% probability level.

**Fig. 2.**
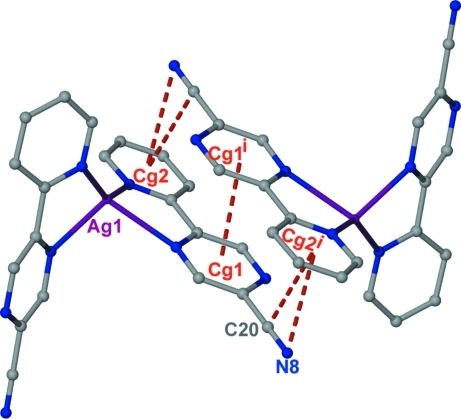
The C≡N···π(pyridyl) and π–π interactions between the mononuclear units, forming the dimer in the title complex.

**Fig. 3.**
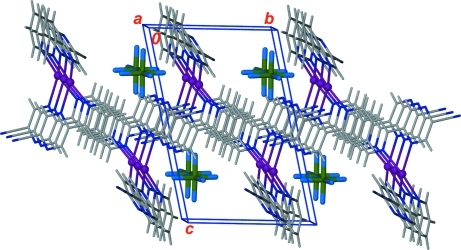
The packing structure of the title complex viewed down the a direction, a layer formed in the ab plane. All non-covalent interactions are omitted for clarity.

## supplementary crystallographic information

### Comment

The coordination chemistry of pyridyl based ligands has intensively developed in the passed decades (Boudalis *et al.*, 2003; Dunne *et al.*, 1997; Wang *et al.*,2009).The devious rigid and/or flexible pyridyl based ligands were designed and synthesized to construct many fancy coordination frameworks (Huang *et al.* 2007). Herein, we report the structure of one new silver(I) complex ([Ag(C_10_H_6_N_4_)_2_]PF_6_) derived from 5-(2-pyridyl)pyrazine-2-carbonitrile, a rigid ligand featuring a 2-cyanopyrazinyl group at the 2-pyridyl carbon atom (Scheme 1).

As shown in Fig.1,two κ^2^ N:N chelating 5-(2-pyridyl)pyrazine-2-carbonitrile ligands surround the Ag^I^ center to form a distorted *N*4-tetrahedral coordination geometry. The Ag—N bond lengthes lie within the range of 2.289 (2)-2.472 (2)Å, with the Ag1—N5 being slight longer than the others, comparable to these in [Ag(C_10_H_6_N_4_)_2_]BF_4_ (2.196 (2)-2.685 (2) Å), a similar complex of 5-(2-pyridyl)pyrazine-2-carbonitrile reported (Wang *et al.* 2010). The two hetero rings of one 5-(2-pyridyl)pyrazine-2-carbonitrile exhibit a dihedral angle of 29.07 (1)°, while in the other one ligand the value is 5.50 (1)°. Such two chelating ligands are almost in an orthogonal orientation, which is remarkablely different from that an anti-relationship in [Ag(C_10_H_6_N_4_)_2_]BF_4_ reported by us (Wang *et al.* 2010). Two mononuclear units arranged in an invert center are interconnected through π(pyrazinyl)···π(pyrazinyl) (Cg1···Cg1^i^ = 3.453 (2) Å, Cg1 = N5-C16-C17-N6-C19-C18 ring, i: -x+1, -y+2, -z+1) and C20≡N8(cyano)···π(pyridyl) interactions (Table 2) to form a dimeric unit. Along the *a* axis, the dimeric units are stacked and interconnected via C11—H11A···N7^iv^(cyano) interaction (Table 1), leading to a column motif. Along the [010] direction, the column motifs interconnect through π(pyridyl)···π(pyridyl) (Cg2···Cg2^ii^ = 3.801 (2) Å, Cg2 = N4-C11-C12-C13-C14-C15 ring, ii: -x+1, -y+1, -z+1) interaction (Table 2), forming a lay in the *ab*plane. Along [001] direction, the formed layers are stacked, and π(pyridyl)···π(pyridyl) interactions (Cg3···Cg3^iii^ = 3.979 (3) Å, Cg3 = N1-C1-C2-C3-C4-C5, iii -x+1, -x+2, -x+2) occur to help to stablize the whole supramolecular structure with the hexafluorophosphate embedded within the interstices.

### Experimental

The ligand 5-(2-pyridyl)-2-cyanopyrazine ligand was obtained commercially. The ligand (18.1 mg, 0.2 mmol) and AgPF_6_ (26 mg, 0.1mmol) were mixed and dissolved in 5 ml solvent of  methanol (3 ml) and acetonitrile (2 ml). After stirring at room temperature for 4 hours, the resulted solution was filtrated, and the clear solution was kepted in air for slow evaporation. After about one week, the colorless block-like crystals were deposited (32.7 mg, 53% yeild).

### Refinement

All the H atoms were discernible in the difference electron density maps. Nevertheless, the hydrogen atoms were placed into idealized positions and allowed to ride on the carrier atoms, with C—H = 0.93 Å and *U*_iso_(H) = 1.2*U*_eq_(C).

### Crystal data

**Table:**

[Ag(C_10_H_6_N_4_)_2_]PF_6_	*Z* = 2
*M**_r_* = 617.22	*F*(000) = 608
Triclinic, *P*1	*D*_x_ = 1.839 Mg m^−^^3^
*a* = 8.8989 (9) Å	Mo *K*α radiation, λ = 0.71073 Å
*b* = 9.1711 (10) Å	Cell parameters from 252 reflections
*c* = 14.0804 (15) Å	θ = 2.3–28.3°
α = 77.023 (2)°	µ = 1.05 mm^−^^1^
β = 86.926 (2)°	*T* = 293 K
γ = 84.809 (2)°	Block, colorless
*V* = 1114.5 (2) Å^3^	0.38 × 0.30 × 0.30 mm

### Data collection

**Table:**

Bruker APEXII CCD area-detector diffractometer	5438 independent reflections
Radiation source: fine-focus sealed tube	4544 reflections with *I* > 2σ(*I*)
graphite	*R*_int_ = 0.017
ω scans	θ_max_ = 28.3°, θ_min_ = 2.3°
Absorption correction: multi-scan (*SADABS*; Bruker, 2007)	*h* = −11→11
*T*_min_ = 0.861, *T*_max_ = 1.000	*k* = −12→11
8106 measured reflections	*l* = −14→18

### Refinement

**Table:**

Refinement on *F*^2^	Primary atom site location: structure-invariant direct methods
Least-squares matrix: full	Secondary atom site location: difference Fourier map
*R*[*F*^2^ > 2σ(*F*^2^)] = 0.032	Hydrogen site location: inferred from neighbouring sites
*wR*(*F*^2^) = 0.090	H-atom parameters constrained
*S* = 1.04	*w* = 1/[σ^2^(*F*_o_^2^) + (0.0427*P*)^2^ + 0.4779*P*] *P* = (*F*_o_^2^ + 2*F*_c_^2^)/3
5438 reflections	(Δ/σ)_max_ = 0.001
325 parameters	Δρ_max_ = 0.71 e Å^−^^3^
0 restraints	Δρ_min_ = −0.37 e Å^−^^3^

### Special details

**Table:**

Geometry. All esds (except the esd in the dihedral angle between two l.s. planes) are estimated using the full covariance matrix. The cell esds are taken into account individually in the estimation of esds in distances, angles and torsion angles; correlations between esds in cell parameters are only used when they are defined by crystal symmetry. An approximate (isotropic) treatment of cell esds is used for estimating esds involving l.s. planes.
Refinement. Refinement of F^2^ against ALL reflections. The weighted R-factor wR and goodness of fit S are based on F^2^, conventional R-factors R are based on F, with F set to zero for negative F^2^. The threshold expression of F^2^ > 2sigma(F^2^) is used only for calculating R-factors(gt) etc. and is not relevant to the choice of reflections for refinement. R-factors based on F^2^ are statistically about twice as large as those based on F, and R- factors based on ALL data will be even larger.

### Fractional atomic coordinates and isotropic or equivalent isotropic displacement parameters (Å^2^)

**Table:**

	*x*	*y*	*z*	*U*_iso_*/*U*_eq_	
Ag1	0.48500 (2)	0.72149 (2)	0.719902 (13)	0.05876 (9)	
N1	0.5186 (2)	0.8140 (2)	0.85560 (14)	0.0472 (4)	
N2	0.2770 (2)	0.6575 (2)	0.83133 (14)	0.0488 (4)	
N3	0.0214 (2)	0.6780 (2)	0.95255 (15)	0.0519 (5)	
N4	0.6022 (2)	0.6137 (2)	0.60099 (14)	0.0421 (4)	
N5	0.3774 (2)	0.8372 (2)	0.55962 (13)	0.0417 (4)	
N6	0.3283 (2)	0.9988 (2)	0.37004 (15)	0.0512 (5)	
N7	−0.2337 (3)	0.5344 (3)	0.8368 (2)	0.0701 (7)	
N8	0.0293 (3)	1.2382 (3)	0.4069 (2)	0.0654 (6)	
C1	0.6476 (3)	0.8679 (3)	0.8722 (2)	0.0577 (6)	
H1A	0.7109	0.9045	0.8191	0.069*	
C2	0.6908 (3)	0.8716 (4)	0.9636 (2)	0.0644 (7)	
H2A	0.7806	0.9108	0.9722	0.077*	
C3	0.5984 (4)	0.8163 (4)	1.0420 (2)	0.0747 (9)	
H3A	0.6266	0.8143	1.1049	0.090*	
C4	0.4630 (3)	0.7635 (4)	1.0269 (2)	0.0659 (7)	
H4A	0.3982	0.7273	1.0793	0.079*	
C5	0.4258 (3)	0.7655 (3)	0.93238 (17)	0.0471 (5)	
C6	0.2814 (3)	0.7132 (3)	0.91130 (15)	0.0429 (5)	
C7	0.1518 (3)	0.7245 (3)	0.97008 (17)	0.0494 (5)	
H7A	0.1570	0.7666	1.0239	0.059*	
C8	0.1461 (3)	0.6118 (3)	0.81258 (18)	0.0526 (6)	
H8A	0.1394	0.5724	0.7577	0.063*	
C9	0.0207 (3)	0.6218 (3)	0.87300 (18)	0.0478 (5)	
C10	−0.1216 (3)	0.5724 (3)	0.8523 (2)	0.0555 (6)	
C11	0.7058 (3)	0.4967 (3)	0.62202 (18)	0.0493 (5)	
H11A	0.7194	0.4500	0.6871	0.059*	
C12	0.7931 (3)	0.4422 (3)	0.5521 (2)	0.0531 (6)	
H12A	0.8631	0.3601	0.5696	0.064*	
C13	0.7749 (3)	0.5111 (3)	0.4566 (2)	0.0551 (6)	
H13A	0.8345	0.4784	0.4080	0.066*	
C14	0.6665 (3)	0.6305 (3)	0.43296 (18)	0.0474 (5)	
H14A	0.6512	0.6779	0.3681	0.057*	
C15	0.5814 (2)	0.6782 (2)	0.50681 (15)	0.0346 (4)	
C16	0.4612 (2)	0.8042 (2)	0.48494 (15)	0.0345 (4)	
C17	0.4369 (3)	0.8876 (3)	0.39062 (17)	0.0487 (5)	
H17A	0.4991	0.8647	0.3398	0.058*	
C18	0.2663 (2)	0.9470 (3)	0.53981 (18)	0.0453 (5)	
H18A	0.2047	0.9712	0.5904	0.054*	
C19	0.2421 (2)	1.0246 (2)	0.44564 (17)	0.0407 (4)	
C20	0.1224 (3)	1.1448 (3)	0.4237 (2)	0.0494 (5)	
P1	0.04799 (7)	1.12551 (7)	0.76794 (5)	0.04787 (15)	
F5	0.0408 (3)	1.1620 (2)	0.65229 (13)	0.0903 (6)	
F4	0.2221 (2)	1.0737 (3)	0.75732 (16)	0.0945 (7)	
F3	−0.1266 (2)	1.1778 (3)	0.77714 (17)	0.0920 (6)	
F6	0.0579 (3)	1.0844 (3)	0.88231 (14)	0.1011 (7)	
F1	0.0880 (3)	1.2901 (2)	0.7634 (2)	0.1208 (9)	
F2	0.0083 (3)	0.9602 (2)	0.77003 (17)	0.0918 (6)	

### Atomic displacement parameters (Å^2^)

**Table:**

	*U*^11^	*U*^22^	*U*^33^	*U*^12^	*U*^13^	*U*^23^
Ag1	0.06857 (14)	0.07215 (15)	0.03810 (11)	−0.00412 (10)	0.00886 (8)	−0.02065 (9)
N1	0.0422 (10)	0.0584 (12)	0.0420 (10)	0.0000 (8)	0.0002 (8)	−0.0152 (9)
N2	0.0525 (11)	0.0576 (12)	0.0382 (10)	−0.0042 (9)	0.0012 (8)	−0.0152 (8)
N3	0.0503 (11)	0.0603 (12)	0.0461 (11)	−0.0050 (9)	0.0022 (9)	−0.0146 (9)
N4	0.0423 (9)	0.0452 (10)	0.0400 (9)	0.0016 (8)	−0.0038 (7)	−0.0136 (8)
N5	0.0372 (9)	0.0492 (10)	0.0403 (9)	0.0015 (7)	0.0019 (7)	−0.0156 (8)
N6	0.0467 (10)	0.0569 (12)	0.0464 (11)	0.0084 (9)	−0.0023 (8)	−0.0088 (9)
N7	0.0617 (14)	0.0812 (17)	0.0726 (16)	−0.0124 (13)	−0.0100 (12)	−0.0234 (13)
N8	0.0524 (12)	0.0628 (14)	0.0816 (17)	0.0149 (11)	−0.0132 (12)	−0.0223 (12)
C1	0.0458 (13)	0.0699 (17)	0.0585 (15)	−0.0028 (12)	0.0013 (11)	−0.0181 (13)
C2	0.0472 (13)	0.083 (2)	0.0680 (18)	−0.0040 (13)	−0.0085 (12)	−0.0263 (15)
C3	0.0706 (18)	0.109 (3)	0.0515 (16)	−0.0126 (17)	−0.0169 (14)	−0.0258 (16)
C4	0.0655 (16)	0.096 (2)	0.0383 (13)	−0.0135 (15)	−0.0038 (11)	−0.0151 (13)
C5	0.0469 (12)	0.0547 (13)	0.0395 (11)	−0.0005 (10)	−0.0023 (9)	−0.0114 (10)
C6	0.0481 (11)	0.0473 (12)	0.0322 (10)	−0.0012 (9)	−0.0012 (8)	−0.0071 (9)
C7	0.0528 (13)	0.0593 (14)	0.0382 (11)	−0.0051 (11)	0.0016 (9)	−0.0156 (10)
C8	0.0591 (14)	0.0607 (14)	0.0415 (12)	−0.0079 (11)	−0.0018 (10)	−0.0175 (11)
C9	0.0507 (12)	0.0477 (12)	0.0437 (12)	−0.0036 (10)	−0.0045 (10)	−0.0064 (10)
C10	0.0570 (15)	0.0587 (15)	0.0513 (14)	−0.0068 (12)	−0.0052 (11)	−0.0116 (11)
C11	0.0507 (13)	0.0499 (13)	0.0471 (13)	0.0053 (10)	−0.0126 (10)	−0.0114 (10)
C12	0.0461 (12)	0.0479 (13)	0.0665 (16)	0.0111 (10)	−0.0114 (11)	−0.0189 (12)
C13	0.0518 (13)	0.0567 (14)	0.0570 (15)	0.0116 (11)	0.0060 (11)	−0.0214 (12)
C14	0.0487 (12)	0.0505 (13)	0.0420 (12)	0.0065 (10)	0.0043 (9)	−0.0134 (10)
C15	0.0311 (9)	0.0349 (9)	0.0395 (10)	−0.0028 (7)	0.0001 (7)	−0.0121 (8)
C16	0.0302 (9)	0.0368 (10)	0.0389 (10)	−0.0037 (7)	0.0001 (7)	−0.0137 (8)
C17	0.0461 (12)	0.0562 (13)	0.0402 (12)	0.0100 (10)	0.0041 (9)	−0.0098 (10)
C18	0.0372 (10)	0.0525 (13)	0.0480 (12)	0.0043 (9)	0.0032 (9)	−0.0194 (10)
C19	0.0310 (9)	0.0394 (10)	0.0540 (13)	−0.0015 (8)	−0.0045 (8)	−0.0148 (9)
C20	0.0384 (11)	0.0507 (13)	0.0610 (15)	0.0020 (10)	−0.0063 (10)	−0.0176 (11)
P1	0.0530 (3)	0.0482 (3)	0.0428 (3)	−0.0012 (3)	0.0021 (2)	−0.0128 (3)
F5	0.1156 (16)	0.0957 (14)	0.0469 (9)	0.0363 (12)	−0.0066 (10)	−0.0060 (9)
F4	0.0560 (10)	0.1330 (19)	0.0913 (15)	0.0151 (11)	−0.0017 (10)	−0.0271 (13)
F3	0.0626 (11)	0.1042 (15)	0.1056 (16)	0.0126 (10)	0.0113 (10)	−0.0262 (13)
F6	0.1187 (17)	0.140 (2)	0.0472 (10)	−0.0087 (15)	−0.0014 (10)	−0.0267 (11)
F1	0.146 (2)	0.0698 (13)	0.160 (2)	−0.0404 (14)	0.0392 (18)	−0.0522 (15)
F2	0.1197 (17)	0.0551 (10)	0.1022 (16)	−0.0126 (10)	−0.0138 (13)	−0.0163 (10)

### Geometric parameters (Å, °)

**Table:**

Ag1—N4	2.2887 (18)	C5—C6	1.478 (3)
Ag1—N1	2.301 (2)	C6—C7	1.393 (3)
Ag1—N2	2.389 (2)	C7—H7A	0.9300
Ag1—N5	2.4714 (19)	C8—C9	1.376 (4)
N1—C5	1.343 (3)	C8—H8A	0.9300
N1—C1	1.342 (3)	C9—C10	1.448 (4)
N2—C8	1.331 (3)	C11—C12	1.374 (4)
N2—C6	1.340 (3)	C11—H11A	0.9300
N3—C7	1.326 (3)	C12—C13	1.362 (4)
N3—C9	1.334 (3)	C12—H12A	0.9300
N4—C15	1.341 (3)	C13—C14	1.386 (3)
N4—C11	1.341 (3)	C13—H13A	0.9300
N5—C16	1.331 (3)	C14—C15	1.381 (3)
N5—C18	1.339 (3)	C14—H14A	0.9300
N6—C19	1.330 (3)	C15—C16	1.492 (3)
N6—C17	1.333 (3)	C16—C17	1.393 (3)
N7—C10	1.134 (4)	C17—H17A	0.9300
N8—C20	1.131 (3)	C18—C19	1.375 (3)
C1—C2	1.371 (4)	C18—H18A	0.9300
C1—H1A	0.9300	C19—C20	1.454 (3)
C2—C3	1.371 (5)	P1—F1	1.568 (2)
C2—H2A	0.9300	P1—F6	1.575 (2)
C3—C4	1.382 (4)	P1—F2	1.5812 (19)
C3—H3A	0.9300	P1—F4	1.5883 (19)
C4—C5	1.385 (3)	P1—F5	1.5906 (18)
C4—H4A	0.9300	P1—F3	1.5908 (19)
			
N4—Ag1—N1	145.43 (7)	C8—C9—C10	121.0 (2)
N4—Ag1—N2	133.18 (7)	N7—C10—C9	179.3 (3)
N1—Ag1—N2	72.30 (7)	N4—C11—C12	123.3 (2)
N4—Ag1—N5	69.52 (6)	N4—C11—H11A	118.4
N1—Ag1—N5	132.63 (7)	C12—C11—H11A	118.4
N2—Ag1—N5	106.77 (7)	C13—C12—C11	118.6 (2)
C5—N1—C1	118.0 (2)	C13—C12—H12A	120.7
C5—N1—Ag1	115.17 (16)	C11—C12—H12A	120.7
C1—N1—Ag1	123.14 (16)	C12—C13—C14	119.1 (2)
C8—N2—C6	117.4 (2)	C12—C13—H13A	120.4
C8—N2—Ag1	127.48 (16)	C14—C13—H13A	120.4
C6—N2—Ag1	112.57 (15)	C15—C14—C13	119.2 (2)
C7—N3—C9	115.6 (2)	C15—C14—H14A	120.4
C15—N4—C11	117.90 (19)	C13—C14—H14A	120.4
C15—N4—Ag1	119.88 (13)	N4—C15—C14	121.82 (19)
C11—N4—Ag1	121.75 (15)	N4—C15—C16	117.00 (17)
C16—N5—C18	117.68 (19)	C14—C15—C16	121.18 (19)
C16—N5—Ag1	113.32 (13)	N5—C16—C17	120.12 (19)
C18—N5—Ag1	127.61 (14)	N5—C16—C15	117.60 (18)
C19—N6—C17	115.7 (2)	C17—C16—C15	122.27 (18)
N1—C1—C2	123.2 (3)	N6—C17—C16	122.8 (2)
N1—C1—H1A	118.4	N6—C17—H17A	118.6
C2—C1—H1A	118.4	C16—C17—H17A	118.6
C1—C2—C3	118.5 (3)	N5—C18—C19	120.9 (2)
C1—C2—H2A	120.8	N5—C18—H18A	119.6
C3—C2—H2A	120.8	C19—C18—H18A	119.6
C2—C3—C4	119.6 (3)	N6—C19—C18	122.76 (19)
C2—C3—H3A	120.2	N6—C19—C20	116.0 (2)
C4—C3—H3A	120.2	C18—C19—C20	121.2 (2)
C3—C4—C5	118.7 (3)	N8—C20—C19	179.9 (3)
C3—C4—H4A	120.7	F1—P1—F6	91.37 (15)
C5—C4—H4A	120.7	F1—P1—F2	178.75 (15)
N1—C5—C4	122.0 (2)	F6—P1—F2	89.84 (13)
N1—C5—C6	116.7 (2)	F1—P1—F4	90.26 (15)
C4—C5—C6	121.3 (2)	F6—P1—F4	90.18 (13)
N2—C6—C7	120.2 (2)	F2—P1—F4	89.45 (13)
N2—C6—C5	117.8 (2)	F1—P1—F5	89.97 (15)
C7—C6—C5	121.9 (2)	F6—P1—F5	178.18 (13)
N3—C7—C6	122.9 (2)	F2—P1—F5	88.81 (12)
N3—C7—H7A	118.6	F4—P1—F5	88.57 (11)
C6—C7—H7A	118.6	F1—P1—F3	89.76 (14)
N2—C8—C9	121.1 (2)	F6—P1—F3	90.52 (13)
N2—C8—H8A	119.4	F2—P1—F3	90.52 (13)
C9—C8—H8A	119.4	F4—P1—F3	179.29 (12)
N3—C9—C8	122.9 (2)	F5—P1—F3	90.72 (12)
N3—C9—C10	116.1 (2)		
			
N4—Ag1—N1—C5	−132.25 (17)	C4—C5—C6—C7	29.9 (4)
N2—Ag1—N1—C5	11.13 (16)	C9—N3—C7—C6	−1.4 (4)
N5—Ag1—N1—C5	107.90 (18)	N2—C6—C7—N3	2.1 (4)
N4—Ag1—N1—C1	25.6 (3)	C5—C6—C7—N3	−179.8 (2)
N2—Ag1—N1—C1	169.0 (2)	C6—N2—C8—C9	0.1 (4)
N5—Ag1—N1—C1	−94.3 (2)	Ag1—N2—C8—C9	−160.13 (19)
N4—Ag1—N2—C8	−43.4 (3)	C7—N3—C9—C8	0.2 (4)
N1—Ag1—N2—C8	164.3 (2)	C7—N3—C9—C10	−179.2 (2)
N5—Ag1—N2—C8	34.0 (2)	N2—C8—C9—N3	0.5 (4)
N4—Ag1—N2—C6	155.58 (14)	N2—C8—C9—C10	179.8 (2)
N1—Ag1—N2—C6	3.24 (16)	N3—C9—C10—N7	11 (29)
N5—Ag1—N2—C6	−127.02 (16)	C8—C9—C10—N7	−169 (100)
N1—Ag1—N4—C15	−124.46 (16)	C15—N4—C11—C12	1.3 (4)
N2—Ag1—N4—C15	106.73 (17)	Ag1—N4—C11—C12	−170.83 (19)
N5—Ag1—N4—C15	12.60 (15)	N4—C11—C12—C13	0.7 (4)
N1—Ag1—N4—C11	47.6 (2)	C11—C12—C13—C14	−1.9 (4)
N2—Ag1—N4—C11	−81.3 (2)	C12—C13—C14—C15	1.1 (4)
N5—Ag1—N4—C11	−175.4 (2)	C11—N4—C15—C14	−2.2 (3)
N4—Ag1—N5—C16	−14.09 (14)	Ag1—N4—C15—C14	170.13 (17)
N1—Ag1—N5—C16	134.22 (14)	C11—N4—C15—C16	177.62 (19)
N2—Ag1—N5—C16	−144.66 (14)	Ag1—N4—C15—C16	−10.1 (2)
N4—Ag1—N5—C18	179.8 (2)	C13—C14—C15—N4	1.0 (3)
N1—Ag1—N5—C18	−31.9 (2)	C13—C14—C15—C16	−178.8 (2)
N2—Ag1—N5—C18	49.2 (2)	C18—N5—C16—C17	3.1 (3)
C5—N1—C1—C2	1.9 (4)	Ag1—N5—C16—C17	−164.53 (17)
Ag1—N1—C1—C2	−155.3 (2)	C18—N5—C16—C15	−177.86 (18)
N1—C1—C2—C3	0.7 (5)	Ag1—N5—C16—C15	14.5 (2)
C1—C2—C3—C4	−2.2 (5)	N4—C15—C16—N5	−4.1 (3)
C2—C3—C4—C5	1.2 (5)	C14—C15—C16—N5	175.7 (2)
C1—N1—C5—C4	−3.0 (4)	N4—C15—C16—C17	174.9 (2)
Ag1—N1—C5—C4	156.0 (2)	C14—C15—C16—C17	−5.3 (3)
C1—N1—C5—C6	177.3 (2)	C19—N6—C17—C16	−0.9 (4)
Ag1—N1—C5—C6	−23.7 (3)	N5—C16—C17—N6	−2.1 (4)
C3—C4—C5—N1	1.5 (5)	C15—C16—C17—N6	178.9 (2)
C3—C4—C5—C6	−178.8 (3)	C16—N5—C18—C19	−1.2 (3)
C8—N2—C6—C7	−1.3 (3)	Ag1—N5—C18—C19	164.36 (16)
Ag1—N2—C6—C7	161.78 (18)	C17—N6—C19—C18	2.8 (3)
C8—N2—C6—C5	−179.5 (2)	C17—N6—C19—C20	−179.4 (2)
Ag1—N2—C6—C5	−16.4 (3)	N5—C18—C19—N6	−1.8 (4)
N1—C5—C6—N2	27.7 (3)	N5—C18—C19—C20	−179.5 (2)
C4—C5—C6—N2	−152.0 (3)	N6—C19—C20—N8	−163 (100)
N1—C5—C6—C7	−150.4 (2)	C18—C19—C20—N8	14 (100)

### Hydrogen-bond geometry (Å, °)

**Table:**

*D*—H···*A*	*D*—H	H···*A*	*D*···*A*	*D*—H···*A*
C11—H11A···N7^i^	0.93	2.47	3.201 (2)	135

Symmetry codes: (i) *x*+1, *y*, *z*.

### Table 2 C≡N···π-electron ring interactions

**Table:**

C≡N···Cg	C···Cg (Å)	N···Cg (Å)	sym. code
C20 N8 Cg2	3.509 (3)	3.519 (2)	-x+1,-y+2,-z+1

^*^ Cg2 is the centroids of N4-C11-C12-C13-C14-C15 (pyridyl)
